# Everybody's Page

**Published:** 1892-07-30

**Authors:** 


### Everybody's Page.

A remarkable recovery from drowning is reported from Sheerness. A few days ago a pinnace was upset through coming into contact with the hawsers of one of the war ships which was being swung. One of three bluejackets in the pinnace was thrown under the boat, where he remained till a hole was pierced through the bottom of the craft, when he was rescued alive. It is not stated how long he was immersed in the water, but it must have been rather an unusual time.

Woman is disputing man's supremacy in every field. In literature, or at least one branch of it, novel writing, she has for long held her own, and now she is claiming her position in the world of science. An "emergency dress" in case of fire, made of asbestos, has been invented by one of the fair sex, which is to do away with the need of a fire escape. Unfortunately, even an "emergency dress" will not do away with the danger of suffocation, which is the cause of so many unfortunate people being overtaken in a fire.

There is, no doubt, much need for restricting the sale of intoxicating liquors in some parts of California, but the action taken by the City Council of Pomona, a city not far from Los Angeles, is of a nature to be expected in an autocratic country, though hardly in a "free" one. Such action here would be looked upon justly as a gross infringement of the liberty of the subject. The Council is not satisfied with inflicting five days' imprisonment, or a heavy fine on any person found with liquor or vessels for holding liquor (!) on his or her person, but gives policemen permission to enter and search houses at any time. Pomona need not be afraid of attracting an extravagant crowd of emigrants.

Judging by recent correspondence, there is plenty of ground available for the propagation of choleraic and other diseases in and around London. According to one unfortunate inhabitant of Shepherd's Bush, Mr. Rudyard Kipling's "Smell of Asia" is completely outdone by the perfume which pervades his district. The receipt for the manufacture of this "Eau de Shepherd's Bush" is alleged by the complainant to be as follows: "Between Shepherd's Bush and Bedford Park there is a piece of waste land, in which a large and deep gravel pit has been dug in recent years, greatly to the profit of the owner. By degrees this pit has been filling with stagnant water, and . . . quantities of parish refuse have been shot into it, so that it is computed there are now in the pond so formed about a million gallons of the most foul-smelling compound imaginable." It is stated that Shepherd's Bush has not a monopoly of the smell, which distributes its favours impartially amongst the inhabitants of Acton, Turnham Green, and Bedford Park also. Even scent gratis is not always acceptable.

"The Chairman of the Great Northern Central Hospital" writes: At the opening ceremony of the bazaar, recently held at Hampstead, in aid of the building fund of the Great Northern Central Hospital, it was announced that a gentleman, who at his own request remains for the present anonymous, had kindly offered to subscribe £500 to the hospital if nine others would follow his example. The Committee have since received two similar promises, and are most anxious to obtain the remaining seven in order to help to meet the expenditure required for the completion of the hospital, which is now in progress. May I most earnestly appeal to the benevolent public to help in this matter? The hospital is doing an excellent work, and the increased accommodation is very urgently needed, the present number of beds being inadequate to meet the wants of the poor in the district.

If the public are beginning to clamour for the regulation of cycle traffic and the taxing of cyclists, the latter have only to thank themselves. As a body, they have for some time been a public nuisance, and now they are becoming a public danger. It is no exaggeration to say that they practically monopolise the roads and country lanes. Why then, it is being very generally asked, should they be exempt from taxation? Why also should not every cyclist have his name and address painted in a conspicuous place on the machine. Now, it is impossible in most cases to obtain any redress from reckless riders; it has been decided in a magisterial court that a policeman has no right forcibly to detain or stop a cyclist, which is tantamount to placing the public at the mercy of these undesirable kings of the road. They certainly make the fullest use of the licence allowed them, which, it is to be hoped, will shortly be curtailed.

The itinerant vendor of ice-cream is a most undesirable importation from every point of view—importation, for he is generally a foreigner. An outbreak of enteric fever in the South-east of London has been traced by the Lewisham Board of Works to the sale of ice-creams by some Italians in Deptford. This discovery has led to representations being made to the London County Council that the sale of such articles should be regulated like that of milk. The Council has, we think, very properly pointed out that the efficient carrying out of the Public Health Act is the available remedy, and that if this were done further provisions would be unnecessary. There are too many useful Acts of Parliament already which have been allowed to become dead letters without adding any more to the same category. There is no reason to suppose that these creams are prepared under conditions other than insanitary, or conditions which will bring their sale within the view of the Public Health Act.

